# Editorial: Professional and scientific societies impacting diversity, equity and inclusion in STEMM

**DOI:** 10.3389/fsoc.2023.1232770

**Published:** 2023-07-04

**Authors:** Verónica A. Segarra, Candice M. Etson

**Affiliations:** ^1^Department of Biological Sciences, Goucher College, Baltimore, MD, United States; ^2^Department of Chemistry, Goucher College, Baltimore, MD, United States; ^3^Alliance to Catalyze Change for Equity in STEM Success, Baltimore, MD, United States; ^4^Leveraging, Enhancing and Developing Biology Research Coordination Network, Rockville, MD, United States; ^5^Department of Molecular Biology and Biochemistry, Wesleyan University, Middletown, CT, United States

**Keywords:** collective impact, equity, diversity, inclusion, professional societies, STEMM

## ProSs and collective impact

Professional and scientific societies (ProSs) are communities brought together by shared expertise, interests, practices, and sometimes even shared identities in the case of identity- or affinity-based ProSs. ProSs can serve as a home community to member scholars and practitioners in different stages of their career in a way that transcends geographical localization and home institution. The ways in which a ProSs serves its membership, its policies, practices, and programming have the potential to sculpt the composition of its membership and ultimately the workforce of the discipline(s) a ProSs represents. As scientists strive to build a global STEMM workforce that is as diverse and as inclusive as it can be, attention has turned to ProSs as possible agents of change toward building a STEMM workforce that is dynamic and representative of the populations and disciplines they represent.

ProSs working toward a diverse and inclusive workforce within their respective disciplines tend to leverage their resources in common ways, and often encounter similar challenges. The emergence of these common patterns motivated five ProSs in the biomedical and life sciences fields to come together in 2017 to establish the Alliance to Catalyze Change for Equity in STEM Success (ACCESS; NSF 1744098). ACCESS was established with the intent to examine and share best practices in areas such as travel awards to annual meetings, speaker selection, and involvement of undergraduate trainees in ProSs activities (Segarra et al., 2020a,b; Etson et al., 2021; Primus et al.). The task of identifying challenges and opportunities proved to be far more effective when sharing data across different ProSs, speaking to the impact that collective work can have on our individual communities.

Collective impact is a developing concept that uses broad cross-sector collaboration in order to achieve large-scale social change and combat the many issues that come with its counterpart, isolated impact (Christens and Inzeo, 2015; Prange et al., 2016; Ennis and Tofa, 2020). Organizations harness collective impact not only to improve the success of their individual goals, but also to welcome new initiatives and ideas that can be integrated to create social change at a larger scale.

## In this Frontiers Research Topic issue

To open the door to collaboration and collective impact, and to engage other ProSs in the conversation, ACCESS has set out to host this Frontiers Research Topic issue. In the sixteen articles that follow, you will find studies and stories that chronicle how ProSs in STEMM are striving to make their membership and scientific communities more diverse and inclusive. These include six articles presenting original research, one describing an educational intervention, two review articles, six perspective articles, and one opinion piece, and they are published in three Frontiers journals—Frontiers in Sociology, Frontiers in Psychology, and Frontiers in Education. This Research Topic of articles represents a variety of voices, including authors at all career stages within academia, as well as many who participate in the scientific endeavor from outside of that structure.

Although it may seem that ProSs are mainly focused on supporting relatively established scholars, the original research articles in this special topic reveal new insights into how participation in ProSs as early as their 1^st^ year of study can support persistence in undergraduate STEMM education for women and members of other underrepresented groups (Smith et al.), not only by providing educational and networking opportunities, but also by increasing their feeling of belonging within the community (Campbell-Montalvo, Kersaint et al.). The importance of this sense of belonging is highlighted by the finding that students with sexual and/or gender minority identities benefit from participating in identity-focused organizations, even if those organizations do not provide as many educational and networking opportunities (Campbell-Montalvo, Cooke et al.). Indeed, many members of groups underrepresented in STEMM experience a conflict between the culture they encounter in educational and professional spaces, and their own identities, attitudes, and beliefs. Much of the research reported as part of this Research Topic explores those conflicts, including work investigating how attitudes and practices in STEMM fields are in conflict with Indigenous people's unique cultural and spiritual perspectives (Ingram et al.).

We believe that ProSs are uniquely situated to make use of findings like those reported in this Research Topic to provide leadership and drive systemic change that will result in standard practices that will be more inclusive. The final two research papers in our topic illustrate that quite well. One is a case study carried out by a small ProSs (the American Elasmobranch Society) representing a deep dive into their own efforts to create a more equitable and inclusive professional society. In addition to their analysis of membership demographics and honest evaluation of an attempted diversity initiative, the authors included a valuable discussion of broad range of potential actions, synthesizing recommendations from a variety of sources, that could be taken by any similar sized ProSs to better support diversity, equity, and inclusion goals (Shiffman et al.). The other describes the impact of a program developed by the Society for Developmental Biology that provided substantive research experiences to undergraduate students who are members of groups underrepresented in STEMM. They found that implementing the program resulted in significant impacts beyond the novice researchers who participated, and helped the society recognize and carry out needed changes in its leadership structure to better represent the needs of its members (Unguez et al.).

The benefits students can receive by having opportunities to participate fully in the community of science during their training period are further highlighted by other articles included in this Research Topic. Readers interested in curriculum intervention may find value in the report on the design and implementation of a biannual student-organized and student-led research conference for students already participating programs providing professional training to members of groups underrepresented in STEMM. This novel intervention goes beyond the typical model of providing coursework and laboratory experience to help students develop the confidence and leadership skills necessary to allow them to envision successful futures in academic science for themselves (Boehmer et al.). Readers looking for more ideas on how ProSs can support their student members may also appreciate the two review articles. One represents a collaboration among six ProSs (including the original five ACCESS ProSs), and presents an examination of the ways these ProSs use society sponsored program offerings to foster inclusivity and engagement of undergraduate scientists (Primus et al.). The other is focused on how student chapters of ProSs can provide students from groups historically underrepresented in STEMM with opportunities to become active members of the ProSs that organize them, at their own pace, as well as to receive the mentoring and support they need (Barnes et al.). In addition, readers will be reminded that the role of student-led organizations should not be discounted, as discussed in a student-authored opinion article (Youngblood et al.).

Rounding out this Research Topic, readers will find articles that share a variety of perspectives on the roles ProSs can play in efforts to reshape the STEMM community. Two provide retrospective reflections on the journeys ProSs have taken along the path to increasing diversity, equity, and inclusion over the lifetime of the organization in one case (Hays et al.), and over the course of a year of concerted effort in another (Segura-Totten et al.). Another perspective article reviews how ProSs report their efforts to support diversity online. In response to their observation that that these materials are often difficult to find, the authors created two webpages gathering them together, providing a valuable resource to our community (Haddad et al.). Yet another shares insights from a group of deaf and hard-of-hearing engineers, scientists, and clinicians on disability as an often overlooked component of diversity (Huyck et al.). The final two perspective articles address the need for tools to facilitate examination of the underlying mental models change leaders may need to address to maximize their ability to effect change (Leibnitz, Gillian-Daniel et al.), and to facilitate self-assessment of diversity, equity, and inclusion efforts at the society level (Leibnitz, Peters et al.).

We hope you will find ideas within these articles that resonate with your own experiences and that can serve as inspiration to continue your work toward a more inclusive and diverse STEMM workforce.

## Author contributions

VAS developed a vision for the editorial and wrote the first draft. CME provided feedback and helped with the revisions. All authors approved the manuscript.
